# Supplementary material for: The Evolution and Characterization of the RNA Interference Pathways in Lophotrochozoa
Source: Genome Biol Evol. 2024 May 7;16(5):evae098. doi: 10.1093/gbe/evae098 (PMC11114477; doi:10.1093/gbe/evae098)
Supplement: evae098_Supplementary_Data [file evae098_supplementary_data.zip › Supplementary_materials.zip!/selected_miRNAs/NoGe_selected_miRNAs/mirdeep_pdf_files/NMRB01003818.1_Notospermus_geniculatus_strain_Ushimado_scaffold3818,_whole_genome_shotgun_sequence_44603.pdf]

Provisional ID	:	NMRB01003818.1_Notospermus_geniculatus_strain_Ushimado_scaffold3818,_whole_genome_shotgun_sequence_44603
Score total	:	106803.8
Score for star read(s)	:	3.9
Score for read counts	:	106795.2
Score for mfe	:	1.7
Score for randfold	:	0
Score for cons. seed	:	3
Total read count	:	209486
Mature read count	:	209327
Loop read count	:	0
Star read count	:	159

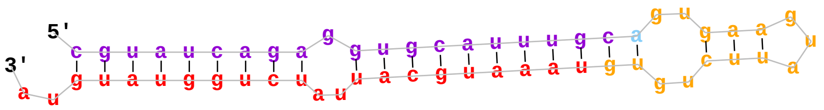

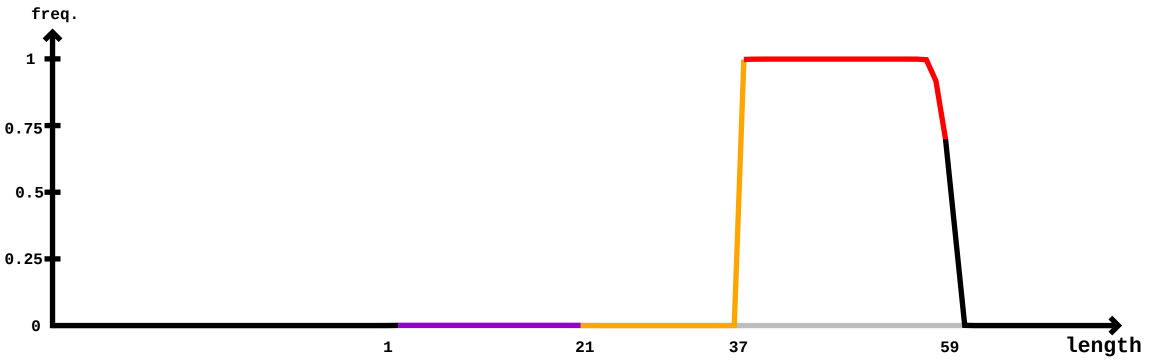


```
                                    Star                              Mature
5'- cuagauguuguuuuguugauaccuguggaggguucguaucagaggugcauuugcagugaaguauucuguguaaaugcauuaucugguauguaacucucgcauguugugc  -3'  obs
    cuagauguuguuuuguugauaccuguggaggguucguaucagaggugcauuugcagugaaguauucuguguaaaugcauuaucugguauguaacucucgcauguugugc       exp
    .(((.(((((......))))).)))..(((((((((((((((((.((((((((((..(((...)))..)))))))))))..))))))))).)))))))((((...))))  reads   mm   sample
    .................................ucguaucagaggugcauuug........................................................  2       0    Fm3
    .................................ucguaucagaggugcauuugc.......................................................  6       0    Fm3
    .................................ucguaucagaggugcauuugca......................................................  3       0    Fm3
    ..................................cguaucagaggugcauuug........................................................  3       0    Fm3
    ..................................cguaucagaggugcauuugc.......................................................  30      0    Fm3
    ..................................Uguaucagaggugcauuugc.......................................................  1       1    Fm3
    ..................................cguaucagaggugcauuCgca.....................................................  1       1    Fm3
    ..................................cguaucagaggugcauuugca.....................................................  15      0    Fm3
    ..................................cguaucagaggugcauGugca.....................................................  1       1    Fm3
    ...................................guaucagaggugcauuugc.......................................................  2       0    Fm3
    .....................................................................Cuaaaugcauuaucugguaug...................  2       1    Fm3
    .....................................................................Cuaaaugcauuaucugguaugu..................  5       1    Fm3
    .....................................................................Cuaaaugcauuaucugguaugua.................  11      1    Fm3
    ......................................................................uaaaugcauuaucugguG.....................  1       1    Fm3
    ......................................................................uaaaugcauuaucuggua.....................  22      0    Fm3
    ......................................................................uaaaugcauuaucugguU.....................  6       1    Fm3
    ......................................................................uaaaugcGuuaucugguau....................  1       1    Fm3
    ......................................................................uaaaugcauuaucugUuau....................  1       1    Fm3
    ......................................................................uaaaugcauuaucugguau....................  212     0    Fm3
    ......................................................................uaaaugcauuaucuggAau....................  1       1    Fm3
    ......................................................................uaaaugcauuCucugguau....................  2       1    Fm3
    ......................................................................uaaaugcauuaucuggCau....................  1       1    Fm3
    ......................................................................uaaCugcauuaucugguau....................  1       1    Fm3
    ......................................................................uaaaugcauuGucugguau....................  1       1    Fm3
    ......................................................................uaaaugcauuaucugguaA....................  7       1    Fm3
    ......................................................................Naaaugcauuaucugguaug...................  5       1    Fm3
    ......................................................................uaaauNcauuaucugguaug...................  3       1    Fm3
    ......................................................................uaaaCgcauuaucugguaug...................  5       1    Fm3
    ......................................................................uaaaugcauuaucuAguaug...................  2       1    Fm3
    ......................................................................uaaaugcauuaucuggGaug...................  24      1    Fm3
    ......................................................................uaaaugcauuaAcugguaug...................  2       1    Fm3
    ......................................................................uaaaugcauuaucugguUug...................  35      1    Fm3
    ......................................................................uaaaugcauuaucuUguaug...................  7       1    Fm3
```

Star **Mature**

cuagauguuguuuuguugauaccuguggaggguucguaucagaggugcauuugcagugaaguauucuguguaaaugcauuaucugguauguaacucucgcauguugugc			
....uaaCugcauuaucugguaug...................	1	1	Fm3
....uaaaugcauuaucuggCaug...................	45	1	Fm3
....uaaaAgcauuaucugguaug...................	4	1	Fm3
....uaaauUcauuaucugguaug...................	3	1	Fm3
....uaaaugcauuaucugguGug...................	42	1	Fm3
....uaaaugcauCaucugguaug...................	1	1	Fm3
....uaaaugcauuUucugguaug...................	5	1	Fm3
....uaaaugcauuaucugguCug...................	57	1	Fm3
....uaaaugcauuGucugguaug...................	17	1	Fm3
....uaaaugcauuCucugguaug...................	13	1	Fm3
....uaaaugcauGaucugguaug...................	1	1	Fm3
....uaCaugcauuaucugguaug...................	1	1	Fm3
....uaaaugNauuaucugguaug...................	1	1	Fm3
....uaaaugcauuauUugguaug...................	16	1	Fm3
....uaaaugcauuaucAgguaug...................	3	1	Fm3
....uaaUugcauuaucugguaug...................	4	1	Fm3
....uaaaugcauuaucGgguaug...................	1	1	Fm3
....uaUaugcauuaucugguaug...................	1	1	Fm3
....uaaaugcauuaucugguaug...................	11685	0	Fm3
....uUaaugcauuaucugguaug...................	3	1	Fm3
....uaaaugUauuaucugguaug...................	4	1	Fm3
....uaaaugcauuaucugAuaug...................	1	1	Fm3
....uaaGugcauuaucugguaug...................	3	1	Fm3
....uaaaugAauuaucugguaug...................	1	1	Fm3
....uaaaugcauuaucuggAaug...................	29	1	Fm3
....uaaaugcUuuaucugguaug...................	3	1	Fm3
....uaaaugcauuaucuCguaug...................	2	1	Fm3
....uaaaugcauuaucugguauU...................	9	1	Fm3
....uaaaugcauuaucugguaAg...................	3	1	Fm3
....uaaaugcauuaucugguauA...................	6	1	Fm3
....uaGaugcauuaucugguaug...................	6	1	Fm3
....uaaaugcauuaucugguaCg...................	1	1	Fm3
....uaaaugcGuuaucugguaug...................	2	1	Fm3
....uaaauNcauuaucugguaugu..................	2	1	Fm3
....uaaaugcauuaucugguaugC..................	1	1	Fm3
....uaaaugUauuaucugguaugu..................	6	1	Fm3
....uaaaAgcauuaucugguaugu..................	2	1	Fm3
....uaaaugcauuaucugguCugu..................	117	1	Fm3
....uaGaugcauuaucugguaugu..................	9	1	Fm3
....uaaaugcauuUucugguaugu..................	19	1	Fm3
....uaaaugcauuaucuggGaugu..................	74	1	Fm3
....uaaaugcauuaucuggCaugu..................	107	1	Fm3
....Caaaugcauuaucugguaugu..................	3	1	Fm3
....uaaaugcauuaucCgguaugu..................	5	1	Fm3
....uaaaugcauuaucugguauAu..................	7	1	Fm3
....uaaaugcauuaucugUuaugu..................	4	1	Fm3
....uaaaugcaCuaucugguaugu..................	2	1	Fm3
....uaaaugcauuaucugguauUu..................	1	1	Fm3
....uaaaGgcauuaucugguaugu..................	1	1	Fm3
....uaaaugcauuaAcugguaugu..................	3	1	Fm3
....uaaaugcauuaucugguauCu..................	1	1	Fm3
....uaaaugcauuaucugguaugu..................	26247	0	Fm3
....uaaaugcauuaucugAuaugu..................	3	1	Fm3
....uaaaugcauuaucuAguaugu..................	9	1	Fm3
....uGaaugcauuaucugguaugu..................	5	1	Fm3
....uaaaugcauuaucAgguaugu..................	7	1	Fm3
....uaaaugcauuaucugguaCgu..................	3	1	Fm3
....uaaaugcauuaCcugguaugu..................	4	1	Fm3
....uaaaugcauuaucugCuaugu..................	1	1	Fm3
....uaaaugcUuuaucugguaugu..................	4	1	Fm3
....uaaaugcauuaucugguUugu..................	77	1	Fm3
....uaCaugcauuaucugguaugu..................	1	1	Fm3
....uaaaugcauuauAugguaugu..................	1	1	Fm3
....uaaaugcauuaucGgguaugu..................	2	1	Fm3
....uaaaugcauuaucuUguaugu..................	11	1	Fm3
....uaaaugcauCaucugguaugu..................	5	1	Fm3
....uaaaugcauuaucugguaugA..................	9	1	Fm3
....uaaaugcauuGucugguaugu..................	31	1	Fm3
....uUaaugcauuaucugguaugu..................	6	1	Fm3
....uaUaugcauuaucugguaugu..................	3	1	Fm3

```
                                   Star                                Mature
cuagauguuguuuuguugauaccuguggaggguucguaucagaggugcauuugcagugaaguauucuguguaaaugcauuaucugguauguaacucucgcauguugugc
......................................................................uaaaugAauuaucugguaugu..................    4       1       Fm3
......................................................................uCaaugcauuaucugguaugu..................    3       1       Fm3
......................................................................Aaaaugcauuaucugguaugu..................    2       1       Fm3
......................................................................uaaGugcauuaucugguaugu..................    6       1       Fm3
......................................................................Gaaaugcauuaucugguaugu..................    2       1       Fm3
......................................................................uaNaugcauuaucugguaugu..................    1       1       Fm3
......................................................................uaaaugcaAuaucugguaugu..................    4       1       Fm3
......................................................................uaaauUcauuaucugguaugu..................    2       1       Fm3
......................................................................uaaaugcauuCucugguaugu..................    21      1       Fm3
......................................................................uaaaCgcauuaucugguaugu..................    8       1       Fm3
......................................................................uaaauAcauuaucugguaugu..................    1       1       Fm3
......................................................................uaaaugcGuuaucugguaugu..................    9       1       Fm3
......................................................................uaaaugcauuaucugguaAgu..................    2       1       Fm3
......................................................................uaaaugcauGaucugguaugu..................    3       1       Fm3
......................................................................uaaaugNauuaucugguaugu..................    1       1       Fm3
......................................................................uaaaugcauuauGugguaugu..................    1       1       Fm3
......................................................................uaaaugcauuaucugguaugG..................    1       1       Fm3
......................................................................uaaaugcauuaucugguGugu..................    63      1       Fm3
......................................................................uaaaugcauuauUugguaugu..................    8       1       Fm3
......................................................................Naaaugcauuaucugguaugu..................    11      1       Fm3
......................................................................uaaaugcauuaucuggAaugu..................    67      1       Fm3
......................................................................uaaaugcauuaucuCguaugu..................    3       1       Fm3
......................................................................uaaCugcauuaucugguaugu..................    3       1       Fm3
......................................................................Aaaaugcauuaucugguaugua.................    1       1       Fm3
......................................................................uaaaugcauuGucugguaugua.................    129     1       Fm3
......................................................................uaaaugcauuUucugguaugua.................    57      1       Fm3
......................................................................Caaaugcauuaucugguaugua.................    20      1       Fm3
......................................................................uaaaugcCuuaucugguaugua.................    1       1       Fm3
......................................................................uaCaugcauuaucugguaugua.................    12      1       Fm3
......................................................................uaaaugcauuaucuCguaugua.................    6       1       Fm3
......................................................................uaaaugcauuaucugguaugAa.................    11      1       Fm3
......................................................................uaaauAcauuaucugguaugua.................    7       1       Fm3
......................................................................uaGaugcauuaucugguaugua.................    33      1       Fm3
......................................................................uGaaugcauuaucugguaugua.................    28      1       Fm3
......................................................................uaaaugcauuCucugguaugua.................    69      1       Fm3
......................................................................uaaaugcauuaAcugguaugua.................    3       1       Fm3
......................................................................uaaaugcauuaucugAuaugua.................    12      1       Fm3
......................................................................uaaauNcauuaucugguaugua.................    15      1       Fm3
......................................................................uaUaugcauuaucugguaugua.................    15      1       Fm3
......................................................................uaaaugcauuaucugguaugua.................    87613   0       Fm3
......................................................................Naaaugcauuaucugguaugua.................    54      1       Fm3
......................................................................uaaaugcGuuaucugguaugua.................    18      1       Fm3
......................................................................uaaaugcauCaucugguaugua.................    4       1       Fm3
......................................................................uaaaugcaCuaucugguaugua.................    12      1       Fm3
......................................................................uaaaugcauuaucugUuaugua.................    12      1       Fm3
......................................................................uaaUugcauuaucugguaugua.................    10      1       Fm3
......................................................................uaaaugcauuaucGgguaugua.................    8       1       Fm3
......................................................................uaaaugcauuaucugguauUua.................    14      1       Fm3
......................................................................uaaaugcauuaCcugguaugua.................    6       1       Fm3
......................................................................uaaaugAauuaucugguaugua.................    3       1       Fm3
......................................................................uaaGugcauuaucugguaugua.................    15      1       Fm3
......................................................................uaaaugUauuaucugguaugua.................    17      1       Fm3
......................................................................uaaaugcauuaucuggGaugua.................    279     1       Fm3
......................................................................uaaaugcauuaucugCuaugua.................    8       1       Fm3
......................................................................uaaaugGauuaucugguaugua.................    2       1       Fm3
......................................................................uaaaugcauuaucugguauguC.................    2       1       Fm3
......................................................................uaaaGgcauuaucugguaugua.................    2       1       Fm3
......................................................................uaaaAgcauuaucugguaugua.................    5       1       Fm3
......................................................................uaaaugcauuaucugguaCgua.................    5       1       Fm3
......................................................................uaaaugcauuaucugguGugua.................    321     1       Fm3
......................................................................uaNaugcauuaucugguaugua.................    1       1       Fm3
......................................................................uaaauCcauuaucugguaugua.................    3       1       Fm3
......................................................................uaaaugcaAuaucugguaugua.................    10      1       Fm3
......................................................................uaaCugcauuaucugguaugua.................    9       1       Fm3
......................................................................uaaaugcauuaucAgguaugua.................    19      1       Fm3
......................................................................Gaaaugcauuaucugguaugua.................    1       1       Fm3
......................................................................uaaaugcauuaucugguaGgua.................    3       1       Fm3
......................................................................uaaaugcauuaucugguauCua.................    2       1       Fm3
......................................................................uaaaugcauuaucuggAaugua.................    180     1       Fm3
......................................................................uaaauUcauuaucugguaugua.................    15      1       Fm3
```

Star Mature

```
cuagauguuguuuuguugauaccuguggaggguucguaucagaggugcauuugcagugaaguauucugugaaaugcauuaucugguauguaacucucgcauguugugc
......................................................................uaaaCgcauuaucugguaugua.................   31    1   Fm3
......................................................................uaaaugcauuaucuUguaugua.................   28    1   Fm3
......................................................................uaaaugcauuaucCgguaugua.................   28    1   Fm3
......................................................................uaaaugcauuaucuggCaugua.................   267   1   Fm3
......................................................................uNaaugcauuaucugguaugua.................   1     1   Fm3
......................................................................uaaaugcauuaucuAguaugua.................   20    1   Fm3
......................................................................uaaaugcaGuaucugguaugua.................   3     1   Fm3
......................................................................uaaaugcauuaucugguCugua.................   339   1   Fm3
......................................................................uaaaugcauuaucugguaAgua.................   4     1   Fm3
......................................................................uaaaugcauGaucugguaugua.................   11    1   Fm3
......................................................................uCaaugcauuaucugguaugua.................   12    1   Fm3
......................................................................uaaaugcauuaucugguauguG.................   33    1   Fm3
......................................................................uaaaugcauuaucugguUugua.................   241   1   Fm3
......................................................................uaaaugNauuaucugguaugua.................   2     1   Fm3
......................................................................uaaaugcauAaucugguaugua.................   1     1   Fm3
......................................................................uaaaugcUuuaucugguaugua.................   35    1   Fm3
......................................................................uUaaugcauuaucugguaugua.................   22    1   Fm3
......................................................................uaaaugcauuauUugguaugua.................   22    1   Fm3
......................................................................uaaaugcauuauAugguaugua.................   8     1   Fm3
......................................................................uaaaugcauuaucugguaugCa.................   12    1   Fm3
......................................................................uaaaugcauuaucugguauAua.................   11    1   Fm3
......................................................................uaaaugcauuaucugguauguU.................   865   1   Fm3
......................................................................uaaaugUauuaucugguauguaa................   1     1   Fm3
......................................................................uaaaugcauuaucugguCuguaa................   21    1   Fm3
......................................................................uaaaugcauuaucugguaGguaa................   1     1   Fm3
......................................................................uaaaugcauuaucuggGauguaa................   12    1   Fm3
......................................................................uaaauAcauuaucugguauguaa................   1     1   Fm3
......................................................................uaCaugcauuaucugguauguaa................   1     1   Fm3
......................................................................uaaaugcauuaGcugguauguaa................   2     1   Fm3
......................................................................uaaaugcGuuaucugguauguaa................   1     1   Fm3
......................................................................uaaaAgcauuaucugguauguaa................   1     1   Fm3
......................................................................Naaaugcauuaucugguauguaa................   2     1   Fm3
......................................................................uaaaugcauuaucugguGuguaa................   22    1   Fm3
......................................................................uaaauUcauuaucugguauguaa................   2     1   Fm3
......................................................................uaaaugcauuaucugguUuguaa................   11    1   Fm3
......................................................................uaaaugAauuaucugguauguaa................   1     1   Fm3
......................................................................uaaaugcauuaucugguaAguaa................   1     1   Fm3
......................................................................uaaaugcauuaucCgguauguaa................   1     1   Fm3
......................................................................uaaaugcauuaucugguauguaG................   1     1   Fm3
......................................................................uaaaugcauuaucugguauAuaa................   1     1   Fm3
......................................................................uaaaCgcauuaucugguauguaa................   1     1   Fm3
......................................................................uaaaugcauuaucuggCauguaa................   16    1   Fm3
......................................................................uaaaugcauuaucGgguauguaa................   1     1   Fm3
......................................................................uaaaugcauuaucuggAauguaa................   17    1   Fm3
......................................................................uaaaugcauuaucugguauguaa................   4866  0   Fm3
......................................................................uaaaugcauuaCcugguauguaa................   1     1   Fm3
......................................................................uUaaugcauuaucugguauguaa................   1     1   Fm3
......................................................................uaaaugcUuuaucugguauguaa................   1     1   Fm3
......................................................................uaaaugcauuUucugguauguaa................   4     1   Fm3
......................................................................uaaaugcauuaucAgguauguaa................   2     1   Fm3
......................................................................uaaaugcauuGucugguauguaa................   8     1   Fm3
......................................................................uaaaugcauuaucugguaCguaa................   1     1   Fm3
......................................................................uaaaugcauuaucugguauguGa................   1     1   Fm3
......................................................................uaaaugcauuaucugguauguUa................   5     1   Fm3
......................................................................uCaaugcauuaucugguauguaa................   1     1   Fm3
......................................................................uaaaugcauuaucuUguauguaa................   1     1   Fm3
......................................................................uaaaugNauuaucugguauguaa................   1     1   Fm3
......................................................................uaaaugcauuaucugguauguaC................   1     1   Fm3
......................................................................uaaaugcauuCucugguauguaa................   6     1   Fm3
......................................................................uaaaugcauuaucugUuauguaa................   1     1   Fm3
......................................................................uaaaugcauuaucugguaugCaa................   1     1   Fm3
......................................................................uaaaugcauuaucuAguauguaa................   1     1   Fm3
......................................................................uaaaugcauuaucugguauguaU................   537   1   Fm3
......................................................................uaaaugcauuaucugguauguaaA...............   27    1   Fm3
......................................................................uaaaugcauuaucugguauguaaU...............   51    1   Fm3
......................................................................uaaaugcauuaucugguauguaac...............   28    0   Fm3
......................................................................uaaaugcauuaucugguauguaaUu..............   3     1   Fm3
......................................................................uaaaugcauuaucugguauguaaAu..............   1     1   Fm3
.......................................................................aaaugcauuaucugguau....................   1     0   Fm3
.......................................................................aaaugcauuaucugguaug...................   12    0   Fm3
```

Star Mature

```
cuagauguuguuuuguugauaccuguggaggguucguaucagaggugcauuugcagugaaguauucuguguaaaugcauuaucugguauguaacucucgcauguugugc
.......................................................................aaaugAauuaucugguaug................... 1 1 Fm3
.......................................................................aaaugcauuaucugguaugu.................. 33 0 Fm3
.......................................................................aaaugcauuCucugguaugu.................. 1 1 Fm3
.......................................................................Uaaugcauuaucugguaugu.................. 7 1 Fm3
.......................................................................aaaugcauuauGugguaugu.................. 1 1 Fm3
.......................................................................aaaugcauuaucugguaugua................. 71 0 Fm3
.......................................................................aaaugcauuaGcugguaugua................. 2 1 Fm3
.......................................................................Uaaugcauuaucugguaugua................. 13 1 Fm3
.......................................................................aaaugcauuaucugguUugua................. 1 1 Fm3
.......................................................................aaaugcauuaucugguauguaa................ 4 0 Fm3
.......................................................................Uaaugcauuaucugguauguaa................ 1 1 Fm3
........................................................................aaugcauuaucugguaug................... 5 0 Fm3
........................................................................aaugcauuaucugguaCgu.................. 1 1 Fm3
........................................................................aaugcauuaucugguaugu.................. 2 0 Fm3
........................................................................aaugcauuaucugguaugua................. 10 0 Fm3
........................................................................aaugcauuaucugguauguaa................ 6 0 Fm3
.........................................................................augcauuaucugguaugu.................. 13 0 Fm3
.........................................................................augcauuaucugguaugua................. 2 0 Fm3
..........................................................................ugcauuaucugguaugua................. 3 0 Fm3

.................................ucguaucagaggugcauuugc....................................................... 4 0 Mm3
..................................cguaucagaggugcauuGgc....................................................... 1 1 Mm3
..................................cguaucagaggugcauuugc....................................................... 1 0 Mm3
..................................cguaucagaggugcauuCgca...................................................... 1 1 Mm3
..................................cguaucagaggugcauuugca...................................................... 6 0 Mm3
..................................cguaucagaggugcauuugcagugaagu............................................... 1 0 Mm3
.....................................................................Cuaaaugcauuaucugguaugu.................. 2 1 Mm3
......................................................................uaaaugcauuaucugguC..................... 1 1 Mm3
......................................................................uaaaugcauuaucuggua..................... 6 0 Mm3
......................................................................uaaaugcauuaucugguau.................... 38 0 Mm3
......................................................................uaaaugcauuaucuggGaug................... 1 1 Mm3
......................................................................uaaaugcauuaucugguaug................... 415 0 Mm3
......................................................................uaaaugcauuaucugguauU................... 2 1 Mm3
......................................................................uaaaugcauuaucugguCug................... 2 1 Mm3
......................................................................uaaaugcauuaucugAuaug................... 1 1 Mm3
......................................................................uaaGugcauuaucugguaug................... 1 1 Mm3
......................................................................uaaaugcauuaucuggCaug................... 5 1 Mm3
......................................................................uaaaugcauuaucugguGug................... 2 1 Mm3
......................................................................uaaaugcauuaucCgguaugu.................. 1 1 Mm3
......................................................................uaaaugcauuaucuggCaugu.................. 5 1 Mm3
......................................................................uaUaugcauuaucugguaugu.................. 1 1 Mm3
......................................................................uaaaugcauuGucugguaugu.................. 1 1 Mm3
......................................................................uaaaugcauuaucugguGugu.................. 7 1 Mm3
......................................................................uaaaugNauuaucugguaugu.................. 1 1 Mm3
......................................................................uaaaugcauuaucugUuaugu.................. 1 1 Mm3
......................................................................uaaaugcauuaucuggAaugu.................. 8 1 Mm3
......................................................................uaaaugcauuaucugguaugC.................. 2 1 Mm3
......................................................................uaaaugcauuaucugguCugu.................. 10 1 Mm3
......................................................................uaaaugcauuaucugguauUu.................. 1 1 Mm3
......................................................................Naaaugcauuaucugguaugu.................. 1 1 Mm3
......................................................................uaaGugcauuaucugguaugu.................. 1 1 Mm3
......................................................................uaaaugcauuaucuUguaugu.................. 1 1 Mm3
......................................................................uaaaugcCuuaucugguaugu.................. 1 1 Mm3
......................................................................uaaaugcauuUucugguaugu.................. 2 1 Mm3
......................................................................uaaauUcauuaucugguaugu.................. 1 1 Mm3
......................................................................uaaaugcauuaucAgguaugu.................. 1 1 Mm3
......................................................................uaaaugcauuaucugguaugG.................. 13 1 Mm3
......................................................................uaaaugcaCuaucugguaugu.................. 1 1 Mm3
......................................................................uaaaugcauuaucugguUugu.................. 7 1 Mm3
......................................................................uaaaugcauuaAcugguaugu.................. 1 1 Mm3
......................................................................uaaaugcauuaucugguaugu.................. 2669 0 Mm3
......................................................................uaaaugcauuaucuggGaugu.................. 3 1 Mm3
......................................................................uUaaugcauuaucugguaugu.................. 1 1 Mm3
......................................................................uaaaugUauuaucugguaugu.................. 1 1 Mm3
......................................................................uaaCugcauuaucugguaugu.................. 1 1 Mm3
......................................................................uaaaugcauAaucugguaugu.................. 1 1 Mm3
......................................................................uaaaugcauuCucugguaugu.................. 2 1 Mm3
......................................................................uaaaAgcauuaucugguaugua................. 1 1 Mm3
......................................................................uaaCugcauuaucugguaugua................. 1 1 Mm3
......................................................................uaaaugcauuaucugAuaugua................. 1 1 Mm3
```

Star **Mature**

```
cuagauguuguuuuguugauaccuguggagguuccguaucagaggugcauuugcagugaaguauucuguguaaaugcauuaucugguauguaacucucgcauguugugc
......................................................................uaaaugcauuaucugguaugAa.................  2     1  Mm3
......................................................................uaaaugcauAaucugguaugua.................  3     1  Mm3
......................................................................uaaaugcauuUucugguaugua.................  3     1  Mm3
......................................................................uUaaugcauuaucugguaugua.................  3     1  Mm3
......................................................................uaaaugcauuaucAgguaugua.................  1     1  Mm3
......................................................................uaaaugcauuaucugguauguG.................  6     1  Mm3
......................................................................uaaaugcauuaucugguaugua.................  8395  0  Mm3
......................................................................uaCaugcauuaucugguaugua.................  2     1  Mm3
......................................................................uaaGugcauuaucugguaugua.................  3     1  Mm3
......................................................................uaaaugAauuaucugguaugua.................  1     1  Mm3
......................................................................uaaaugcauuGucugguaugua.................  19    1  Mm3
......................................................................uaaaugcauuaucugguaAgua.................  1     1  Mm3
......................................................................Caaaugcauuaucugguaugua.................  3     1  Mm3
......................................................................uaaaugcauuaucugguauUua.................  2     1  Mm3
......................................................................uaaaugcauuaucugguaugCa.................  14    1  Mm3
......................................................................uaaaugNauuaucugguaugua.................  1     1  Mm3
......................................................................uaaaugcauuaucGgguaugua.................  1     1  Mm3
......................................................................uaUaugcauuaucugguaugua.................  1     1  Mm3
......................................................................Naaaugcauuaucugguaugua.................  6     1  Mm3
......................................................................uGaaugcauuaucugguaugua.................  1     1  Mm3
......................................................................uaaaugcauuaucuggAaugua.................  12    1  Mm3
......................................................................uaaauUcauuaucugguaugua.................  2     1  Mm3
......................................................................uaaaugcauuaucugguauguU.................  104   1  Mm3
......................................................................uaaaugcauuaucugguUugua.................  21    1  Mm3
......................................................................uaaaugcauGaucugguaugua.................  1     1  Mm3
......................................................................uaaaugcauuaucCgguaugua.................  2     1  Mm3
......................................................................uaaaCgcauuaucugguaugua.................  1     1  Mm3
......................................................................uaaaugcauuaucuggCaugua.................  24    1  Mm3
......................................................................uaaaugcauuaucugguauAua.................  1     1  Mm3
......................................................................uaaaugcauuCucugguaugua.................  3     1  Mm3
......................................................................uaaaugcauuaucugguCugua.................  36    1  Mm3
......................................................................uaaaugcUuuaucugguaugua.................  3     1  Mm3
......................................................................uaaauNcauuaucugguaugua.................  1     1  Mm3
......................................................................uaaaugcaAuaucugguaugua.................  1     1  Mm3
......................................................................uaaaugcauuauAugguaugua.................  1     1  Mm3
......................................................................uaaaugcauuaucugUuaugua.................  2     1  Mm3
......................................................................uaaauAcauuaucugguaugua.................  1     1  Mm3
......................................................................uaaaugcauuaucuggGaugua.................  14    1  Mm3
......................................................................uaaaugcauuaucugCuaugua.................  1     1  Mm3
......................................................................uaaUugcauuaucugguaugua.................  4     1  Mm3
......................................................................uaaaugUauuaucugguaugua.................  1     1  Mm3
......................................................................uaaaugcauuaucugguGugua.................  25    1  Mm3
......................................................................uaaaugcauuGucugguauguaa................  1     1  Mm3
......................................................................uaaaugcauuaucugguauguaU................  60    1  Mm3
......................................................................uaaaugcauuUucugguauguaa................  1     1  Mm3
......................................................................uaaaugcauuauUugguauguaa................  1     1  Mm3
......................................................................uaaaugcauuaucugguCuguaa................  2     1  Mm3
......................................................................uaaaugcauuaucugguGuguaa................  1     1  Mm3
......................................................................uaaaugcauuaucugguauguaa................  531   0  Mm3
......................................................................uaaaugNauuaucugguauguaa................  1     1  Mm3
......................................................................Caaaugcauuaucugguauguaa................  1     1  Mm3
......................................................................uaaaugcauuaucuggGauguaa................  2     1  Mm3
......................................................................uaaaugcauuaucugguUuguaa................  2     1  Mm3
......................................................................uaaaugcauuaucuggAauguaa................  1     1  Mm3
......................................................................uaaaugcauuaucugguauguaaA...............  4     1  Mm3
......................................................................uaaaugcauuaucugguauguaac...............  3     0  Mm3
......................................................................uaaaugcauuaucugguauguaaU...............  9     1  Mm3
......................................................................uaaaugcauuaucugguauguaaUu..............  1     1  Mm3
.......................................................................aaaugcauuaucugguaugu..................  2     0  Mm3
.......................................................................aaaugcauuaAcugguaugu..................  1     1  Mm3
.......................................................................aaaugcauuaucugguaugua.................  18    0  Mm3
.......................................................................aaaugcauuaucugguauguaa................  1     0  Mm3
.........................................................................augcauuaucugguaugua.................  1     0  Mm3

.....................................................................Cuaaaugcauuaucugguaugua.................  15    1  Fm2
......................................................................uaaaugcauuaucuggua.....................  7     0  Fm2
......................................................................uaaaugcauuaucugguau....................  37    0  Fm2
......................................................................uaaaugcauuaucuggCau....................  1     1  Fm2
......................................................................uaaaugcauuaucuggCaug...................  9     1  Fm2
......................................................................uaaaugcauuaucuAguaug...................  1     1  Fm2
```

Star Mature

```
cuagauguuguuuuguugauaccuguggagguucguaucagaggugcauuugcagugaaguauucuguguaaaugcauuaucugguauguaacucucgcauguugugc
.....................................................................uaaaGgcauuaucugguaug...................  1     1  Fm2
.....................................................................uaaaugcauuCucugguaug...................  1     1  Fm2
.....................................................................uaaaugcauuaucugguaug...................  1551  0  Fm2
.....................................................................uaaGugcauuaucugguaug...................  1     1  Fm2
.....................................................................uaaaugcauuaucuggAaug...................  2     1  Fm2
.....................................................................uaaaugcGuuaucugguaug...................  1     1  Fm2
.....................................................................uaaaugcauuaCcugguaug...................  1     1  Fm2
.....................................................................uaGaugcauuaucugguaug...................  1     1  Fm2
.....................................................................uaaaugcauuauGugguaug...................  1     1  Fm2
.....................................................................uaaaugcauuaucugguUug...................  2     1  Fm2
.....................................................................uaaaugcauuaucugguCug...................  7     1  Fm2
.....................................................................uaaCugcauuaucugguaug...................  1     1  Fm2
.....................................................................Naaaugcauuaucugguaug...................  2     1  Fm2
.....................................................................uaaaugcauuaucuggGaug...................  2     1  Fm2
.....................................................................uaaaugcauuaucugguGug...................  6     1  Fm2
.....................................................................uaaaugcauCaucugguaug...................  1     1  Fm2
.....................................................................uCaaugcauuaucugguaug...................  1     1  Fm2
.....................................................................uGaaugcauuaucugguaugu..................  2     1  Fm2
.....................................................................Naaaugcauuaucugguaugu..................  2     1  Fm2
.....................................................................uaaUugcauuaucugguaugu..................  1     1  Fm2
.....................................................................uaaauUcauuaucugguaugu..................  1     1  Fm2
.....................................................................uaaaugcaCuaucugguaugu..................  1     1  Fm2
.....................................................................uaCaugcauuaucugguaugu..................  1     1  Fm2
.....................................................................uaaaugcauuUucugguaugu..................  1     1  Fm2
.....................................................................uCaaugcauuaucugguaugu..................  1     1  Fm2
.....................................................................uaaaugcauuGucugguaugu..................  4     1  Fm2
.....................................................................uaaaugcauuaucugUuaugu..................  1     1  Fm2
.....................................................................uaaaugcauuaucugguCugu..................  7     1  Fm2
.....................................................................uaaaugcauuaucuUguaugu..................  2     1  Fm2
.....................................................................uaGaugcauuaucugguaugu..................  1     1  Fm2
.....................................................................uaaaugcauuaucuggCaugu..................  10    1  Fm2
.....................................................................uaaaugcauuaucugguUugu..................  3     1  Fm2
.....................................................................uaaaugAauuaucugguaugu..................  1     1  Fm2
.....................................................................uaaaugcauuaucugguaugG..................  16    1  Fm2
.....................................................................uaaaugcauuaucugguaugu..................  2991  0  Fm2
.....................................................................uaaaugcauuCucugguaugu..................  4     1  Fm2
.....................................................................uaaaugcauuaGcugguaugu..................  1     1  Fm2
.....................................................................uaaaugcauuaucugguGugu..................  5     1  Fm2
.....................................................................uaaaugcauuauUugguaugu..................  1     1  Fm2
.....................................................................uaaaugcUuuaucugguaugu..................  1     1  Fm2
.....................................................................uaaaugcauuaucuggGaugu..................  8     1  Fm2
.....................................................................uaaaugcauuaucuggAaugu..................  4     1  Fm2
.....................................................................uaaauAcauuaucugguaugu..................  1     1  Fm2
.....................................................................uaaaugcauuaucGgguaugua.................  1     1  Fm2
.....................................................................uaaaugcauuaucugguaCgua.................  1     1  Fm2
.....................................................................uaaaugcauuaAcugguaugua.................  2     1  Fm2
.....................................................................uaaaugcauuaucugguauUua.................  2     1  Fm2
.....................................................................uaaaugcauuaGcugguaugua.................  1     1  Fm2
.....................................................................Aaaaugcauuaucugguaugua.................  2     1  Fm2
.....................................................................uaaaugcauuaucCgguaugua.................  1     1  Fm2
.....................................................................uaUaugcauuaucugguaugua.................  2     1  Fm2
.....................................................................uaaaugcaCuaucugguaugua.................  1     1  Fm2
.....................................................................uaGaugcauuaucugguaugua.................  5     1  Fm2
.....................................................................uaaaugcauuaCcugguaugua.................  1     1  Fm2
.....................................................................uaaaugcUuuaucugguaugua.................  4     1  Fm2
.....................................................................uaaaugGauuaucugguaugua.................  2     1  Fm2
.....................................................................uaaaugcauuaucuggAaugua.................  24    1  Fm2
.....................................................................uaaaugcauuaucuAguaugua.................  4     1  Fm2
.....................................................................uaaaugcauuaucugguauCua.................  1     1  Fm2
.....................................................................uCaaugcauuaucugguaugua.................  3     1  Fm2
.....................................................................uaaaAgcauuaucugguaugua.................  1     1  Fm2
.....................................................................uaaaugcaAuaucugguaugua.................  3     1  Fm2
.....................................................................uaaaugcauuaucuggCaugua.................  50    1  Fm2
.....................................................................uaaaugcauuaucugguaAgua.................  1     1  Fm2
.....................................................................uaaauUcauuaucugguaugua.................  2     1  Fm2
.....................................................................uaaUugcauuaucugguaugua.................  3     1  Fm2
.....................................................................uaaaugcGuuaucugguaugua.................  3     1  Fm2
.....................................................................uaaaugcauuaucuggGaugua.................  31    1  Fm2
.....................................................................uaaaugcauGaucugguaugua.................  1     1  Fm2
.....................................................................Naaaugcauuaucugguaugua.................  9     1  Fm2
```

Star Mature

cuagauguuguuuuguugauaccuguggaggguucguaucagaggugcauuugcagugaaguauucugugauaaaugcauuaucugguauguaacucucgcauguugugc			
....uaaCugcauuaucugguaugua.................	2	1	Fm2
....uaaaugUauuaucugguaugua.................	4	1	Fm2
....uaaaugcauuaucugguaugCa.................	4	1	Fm2
....uaaaugcauuaucuCguaugua.................	2	1	Fm2
....uaaaugcauuauUugguaugua.................	2	1	Fm2
....uaaaugcauuaucugUuaugua.................	3	1	Fm2
....uaaaCgcauuaucugguaugua.................	2	1	Fm2
....uaaauNcauuaucugguaugua.................	1	1	Fm2
....uaaaugcauuaucuUguaugua.................	4	1	Fm2
....uaaaugcauuauAugguaugua.................	1	1	Fm2
....uaaaugcauuaucAgguaugua.................	5	1	Fm2
....uaaaugcauCaucugguaugua.................	1	1	Fm2
....uaaaugcauuaucugguauguU.................	116	1	Fm2
....Caaaugcauuaucugguaugua.................	4	1	Fm2
....uaaaugcauuaucugguGugua.................	35	1	Fm2
....uaaaugcauuaucugguauguC.................	2	1	Fm2
....uaaaugcauuUcugguaugua.................	8	1	Fm2
....uaaaugcauuaucugguUugua.................	41	1	Fm2
....uaaaugcauuaucugguauAua.................	2	1	Fm2
....uaaaugcauuaucugguCugua.................	44	1	Fm2
....uaaaugcauuaucugguaugua.................	12108	0	Fm2
....uaaaugcauuCucugguaugua.................	11	1	Fm2
....uaaauAcauuaucugguaugua.................	1	1	Fm2
....uaaaugcauuGucugguaugua.................	9	1	Fm2
....uaaaugcauuaucugguauguG.................	20	1	Fm2
....uaaaugcauuaucugguauguaa................	773	0	Fm2
....uaaaugcauuaucugguUuguaa................	3	1	Fm2
....uaaauNcauuaucugguauguaa................	1	1	Fm2
....uaaaugcauuGucugguauguaa................	1	1	Fm2
....uaaaugcauuaucugguauguaG................	9	1	Fm2
....uaaaugcauuaucuggAauguaa................	2	1	Fm2
....uaaaugcauuaucugguauguaU................	101	1	Fm2
....uaaaugcauuaucugguCuguaa................	1	1	Fm2
....uaaaugcauuaucugguGuguaa................	5	1	Fm2
....uaaaugcauuUcugguauguaa................	3	1	Fm2
....uaaaugcauuaucugguauguaaU...............	17	1	Fm2
....aaaugcauuaucugguaug...................	8	0	Fm2
....aaaugcauuaucugguaugu..................	2	0	Fm2
....aaaugcauuaGcugguaugu..................	1	1	Fm2
....Uaaugcauuaucugguaugua.................	1	1	Fm2
....aaaugcauuaucugguaugua.................	5	0	Fm2
....aaugcauuaucugguaugua.................	4	0	Fm2
....aaugcauuaucugguauguaa................	9	0	Fm2
.................................ucguaucagaggugcauuug..	3	0	Fm1
.................................ucguaucagaggugcauuugc...	9	0	Fm1
..................................cguaucagaggugcauuug..	4	0	Fm1
..................................cguaucagaggugcauuugc...	8	0	Fm1
..................................cguaucagaggugcauuugca..	11	0	Fm1
....Cuaaaugcauuaucugguaugua.................	3	1	Fm1
....uaaaugcauuaucuggua.....................	3	0	Fm1
....uaaaugcauuaucugguU.....................	5	1	Fm1
....uaaaugcauuaucugguau....................	38	0	Fm1
....uaaaugcauuaucugguGu....................	1	1	Fm1
....uaaaugcauuaucugguaA....................	1	1	Fm1
....uaaaugcauuaucuggGau....................	1	1	Fm1
....uaaaugcauuaucugguUu....................	3	1	Fm1
....uaaaugcauuaucuggAaug...................	2	1	Fm1
....uaaaugcauuaucuggGaug...................	2	1	Fm1
....uaaaugcauuaucugguauA...................	1	1	Fm1
....uaaaugcaAuaucugguaug...................	1	1	Fm1
....uaaaugcauuGucugguaug...................	3	1	Fm1
....uaaaugcauuaucuggCaug...................	2	1	Fm1
....uaaaugcauuaucugguGug...................	4	1	Fm1
....uaaaugcauuaucugguCug...................	3	1	Fm1
....uaaauUcauuaucugguaug...................	1	1	Fm1
....uaaaugcauuaucugAuaug...................	1	1	Fm1
....uaaaugcauuaucugguaGg...................	3	1	Fm1
....uaaaugcauuaucugguaug...................	1198	0	Fm1
....uaaaugcGuuaucugguaug...................	1	1	Fm1

```
                                   Star                              Mature
cuagauguuguuuuguugauaccuguggaggguucguaucagaggugcauuugcagugaaguauucugugcaaaugcauuaucugguauguaacucucgcauguugugc
......................................................................uaaaugcauuaucugguauU...................	4	1	Fm1
......................................................................uaGaugcauuaucugguaug...................	1	1	Fm1
......................................................................uaaaugcauuauUgguaug....................	2	1	Fm1
......................................................................uaaaugcauuaucugguUug...................	2	1	Fm1
......................................................................Naaaugcauuaucugguaugu..................	3	1	Fm1
......................................................................uaaaugcauuaucugguauUu..................	2	1	Fm1
......................................................................uaaaugcauuCucugguaugu..................	4	1	Fm1
......................................................................uaaaugcauuaCcugguaugu..................	1	1	Fm1
......................................................................uaaaugcauuauUgguaugu...................	2	1	Fm1
......................................................................uaaaugcauuaucGgguaugu..................	1	1	Fm1
......................................................................uaaaugcauuaucugguUugu..................	7	1	Fm1
......................................................................uaaaugcauuaucugguCugu..................	15	1	Fm1
......................................................................Caaaugcauuaucugguaugu..................	1	1	Fm1
......................................................................uaaaugcauuaucugguaugu..................	3328	0	Fm1
......................................................................uaGaugcauuaucugguaugu..................	1	1	Fm1
......................................................................uaaaugcauuaucugguauAu..................	2	1	Fm1
......................................................................uaaaugcauuaucugguaugG..................	15	1	Fm1
......................................................................uaaCugcauuaucugguaugu..................	1	1	Fm1
......................................................................uaaauUcauuaucugguaugu..................	1	1	Fm1
......................................................................uUaaugcauuaucugguaugu..................	2	1	Fm1
......................................................................uaaaugNauuaucugguaugu..................	1	1	Fm1
......................................................................uaaaugAauuaucugguaugu..................	1	1	Fm1
......................................................................uaaaugcauuaucuggGaugu..................	5	1	Fm1
......................................................................uaaaugcauuGucugguaugu..................	6	1	Fm1
......................................................................uaaUugcauuaucugguaugu..................	2	1	Fm1
......................................................................uaCaugcauuaucugguaugu..................	1	1	Fm1
......................................................................uaaaugcauuaucuggCaugu..................	9	1	Fm1
......................................................................uaaaugcaGuaucugguaugu..................	1	1	Fm1
......................................................................uaaaugcauuaucugguGugu..................	7	1	Fm1
......................................................................uaaaugcauuaucuggAaugu..................	2	1	Fm1
......................................................................uaaaugcauuaucugguaCgu..................	1	1	Fm1
......................................................................uaaaugcUuuaucugguaugu..................	4	1	Fm1
......................................................................uaaaugcauuUucugguaugua.................	9	1	Fm1
......................................................................uaaaugcauuaucugguaugAa.................	3	1	Fm1
......................................................................uaaaugcauuaucugguauguG.................	15	1	Fm1
......................................................................uaaaugcaCuaucugguaugua.................	2	1	Fm1
......................................................................uaaauUcauuaucugguaugua.................	3	1	Fm1
......................................................................uaaaugcauuaucugguaugua.................	12607	0	Fm1
......................................................................uaaaugcauuaucuggCaugua.................	60	1	Fm1
......................................................................uaaaugcauuaucCgguaugua.................	3	1	Fm1
......................................................................Aaaaugcauuaucugguaugua.................	5	1	Fm1
......................................................................uaaaugcauuaucuggAaugua.................	29	1	Fm1
......................................................................uaaaugcauuaucAgguaugua.................	5	1	Fm1
......................................................................uaaaugcauGaucugguaugua.................	3	1	Fm1
......................................................................uaaauAcauuaucugguaugua.................	1	1	Fm1
......................................................................uaaaugcauuaucugguauguU.................	101	1	Fm1
......................................................................uaaaugcGuuaucugguaugua.................	7	1	Fm1
......................................................................uUaaugcauuaucugguaugua.................	7	1	Fm1
......................................................................uaaaugcauuaucugguCugua.................	45	1	Fm1
......................................................................uaaaugcauuaucuCguaugua.................	1	1	Fm1
......................................................................uaaaugcauuaucuggGaugua.................	36	1	Fm1
......................................................................uaaauNcauuaucugguaugua.................	1	1	Fm1
......................................................................uaaaugcauuaucugCuaugua.................	1	1	Fm1
......................................................................uaaaugcauuaCcugguaugua.................	1	1	Fm1
......................................................................uaaaAgcauuaucugguaugua.................	1	1	Fm1
......................................................................uaaaugUauuaucugguaugua.................	2	1	Fm1
......................................................................uaaaugcauuaucugguauguC.................	4	1	Fm1
......................................................................uaaaugGauuaucugguaugua.................	1	1	Fm1
......................................................................uaaaugAauuaucugguaugua.................	1	1	Fm1
......................................................................uaaaugcauuCucugguaugua.................	15	1	Fm1
......................................................................uaaaugcauuaucugguaAgua.................	1	1	Fm1
......................................................................uaaaugcUuuaucugguaugua.................	4	1	Fm1
......................................................................uaaaugcauuaucugguUugua.................	42	1	Fm1
......................................................................uGaaugcauuaucugguaugua.................	1	1	Fm1
......................................................................uaaUugcauuaucugguaugua.................	2	1	Fm1
......................................................................uaaaugcauuGucugguaugua.................	18	1	Fm1
......................................................................uaGaugcauuaucugguaugua.................	4	1	Fm1
......................................................................uaaaugcauuaucugguaugCa.................	8	1	Fm1
......................................................................uaaaugcaGuaucugguaugua.................	1	1	Fm1
......................................................................uaCaugcauuaucugguaugua.................	3	1	Fm1
```

Star Mature

cuagauguuguuuuguugauaccuguggaggguucguaucagaggugcauuugcagugaaguauucugugu aaaugcauuaucugguauguaacucucgcauguugugc			
....uaUaugcauuaucugguaugua.................	1	1	Fm1
....uaaCugcauuaucugguaugua.................	3	1	Fm1
....uaaaugcauuaucugguaGgua.................	1	1	Fm1
....Naaaugcauuaucugguaugua.................	7	1	Fm1
....uaaaCgcauuaucugguaugua.................	1	1	Fm1
....uaaaugcauuaucugUuaugua.................	3	1	Fm1
....uaaaugcauuaucugguauAua.................	4	1	Fm1
....uaaaGgcauuaucugguaugua.................	1	1	Fm1
....Caaaugcauuaucugguaugua.................	11	1	Fm1
....uaaaugcauuaucugguGugua.................	33	1	Fm1
....uaaaugcauuaucuUguaugua.................	1	1	Fm1
....uaaaugcGuuaucugguauguaa................	2	1	Fm1
....uaaaugcauuaucugguCuguaa................	2	1	Fm1
....uaaaugcauuGucugguauguaa................	1	1	Fm1
....uaaaugcauuaucugguauguaU................	137	1	Fm1
....uaaaugcauuaucugguauguaa................	693	0	Fm1
....uGaaugcauuaucugguauguaa................	1	1	Fm1
....uaaaugcauuaucugguauUuaa................	1	1	Fm1
....uaaaugcauuaucugguUuguaa................	3	1	Fm1
....uaaaugcauuaucuggGauguaa................	2	1	Fm1
....uaaaugcauuaucugguGuguaa................	1	1	Fm1
....uaaaugcauuaucugguauguaaG...............	1	1	Fm1
....uaaaugcauuaucugguauguaac...............	2	0	Fm1
....uaaaugcauuaucugguauguaaU...............	9	1	Fm1
....uaaaugcauuaucugguauguaaA...............	2	1	Fm1
....aaaugcauuaucugguaug...................	1	0	Fm1
....aaaugcauuaucugguauUu..................	1	1	Fm1
....aaaugcauuaucugguaugu..................	6	0	Fm1
....Uaaugcauuaucugguaugu..................	1	1	Fm1
....Uaaugcauuaucugguaugua.................	2	1	Fm1
....aaaugcauuaucugguaugua.................	16	0	Fm1
....aaaugcauuaucugguaugAa.................	1	1	Fm1
....aaugcauuaucugguaugua.................	2	0	Fm1
....aaugcauuaucugguauguaa................	1	0	Fm1
..................................cguaucagaggugcauuugU...	1	1	Mm2
..................................cguaucagaggugcauuugca..	3	0	Mm2
....Cuaaaugcauuaucugguaug...................	1	1	Mm2
....uaaaugcauuaucuggua.....................	1	0	Mm2
....uaaaugcauuaucugguau....................	16	0	Mm2
....uaaaugcauuaucugguUug...................	1	1	Mm2
....uaaaugcauuaucugguaug...................	418	0	Mm2
....uaaaugcauuaucuggAaug...................	1	1	Mm2
....uaaaugcauuGucugguaug...................	3	1	Mm2
....uaaaugcauuaucugguGug...................	1	1	Mm2
....uaaaugcauuaucugguCug...................	2	1	Mm2
....uaaaugcauuCucugguaug...................	1	1	Mm2
....uaaaugcauuaucuggCaug...................	1	1	Mm2
....uaaaugcauuUucugguaugu..................	1	1	Mm2
....uaaaugcauuaucugguUugu..................	3	1	Mm2
....uaaaugcauuGucugguaugu..................	5	1	Mm2
....uaaaugcauuaucugguGugu..................	8	1	Mm2
....uaaaugcaCuaucugguaugu..................	1	1	Mm2
....uaaaugcauuaucugguaugG..................	5	1	Mm2
....uaaaugcauuCucugguaugu..................	1	1	Mm2
....uaaaugcUuuaucugguaugu..................	1	1	Mm2
....uaaaugcauuaucugguaugC..................	3	1	Mm2
....uaaaCgcauuaucugguaugu..................	1	1	Mm2
....uaaGugcauuaucugguaugu..................	1	1	Mm2
....uaaaugcauGaucugguaugu..................	1	1	Mm2
....uaaaugcauuaucuggGaugu..................	2	1	Mm2
....uaaaugcauuaucAgguaugu..................	1	1	Mm2
....uaaauAcauuaucugguaugu..................	1	1	Mm2
....uaaaugcauuaucugguCugu..................	6	1	Mm2
....uaGaugcauuaucugguaugu..................	3	1	Mm2
....uaaaugcauuaucuggAaugu..................	4	1	Mm2
....uaaaugcauuaucugguaugu..................	1673	0	Mm2
....uGaaugcauuaucugguaugu..................	1	1	Mm2
....uaaUugcauuaucugguaugu..................	1	1	Mm2
....uaaaugcauuaucuggCaugu..................	12	1	Mm2

```
                                  Star                                  Mature
cuagauguuguuuuguugauaccuguggaggguucguaucagaggugcauuugcagugaaguauucuguguaaaugcauuaucugguauguaacucucgcauguugugc
......................................................................uaaaugcauuaucCgguaugu..................   2     1   Mm2
......................................................................uaGaugcauuaucugguaugua.................   2     1   Mm2
......................................................................uaaaugcauuaucugAuaugua.................   1     1   Mm2
......................................................................uaaaugcaAuaucugguaugua.................   1     1   Mm2
......................................................................uaaaugcauuaucuggCaugua.................   15    1   Mm2
......................................................................uaUaugcauuaucugguaugua.................   2     1   Mm2
......................................................................uaaaugcauuaucugguaGgua.................   1     1   Mm2
......................................................................uaaaugcauuaucuggGaugua.................   7     1   Mm2
......................................................................uaaaGgcauuaucugguaugua.................   1     1   Mm2
......................................................................uaaaugcCuuaucugguaugua.................   1     1   Mm2
......................................................................uaaaugcauuaucugguauguG.................   8     1   Mm2
......................................................................uaaaugcauuaucugguaugua.................   3920  0   Mm2
......................................................................uaaaugcauuCucugguaugua.................   8     1   Mm2
......................................................................Aaaaugcauuaucugguaugua.................   1     1   Mm2
......................................................................uGaaugcauuaucugguaugua.................   2     1   Mm2
......................................................................uUaaugcauuaucugguaugua.................   4     1   Mm2
......................................................................uaaaugcauuaucugguauguU.................   39    1   Mm2
......................................................................uaaaugcauuaucuUguaugua.................   1     1   Mm2
......................................................................uaaaugcauCaucugguaugua.................   1     1   Mm2
......................................................................uaaaugcauuaucuggAaugua.................   9     1   Mm2
......................................................................uaaaugcauuaucugguUugua.................   9     1   Mm2
......................................................................uaaaugcauuGucugguaugua.................   1     1   Mm2
......................................................................uaaaugcauuaucAgguaugua.................   2     1   Mm2
......................................................................Naaaugcauuaucugguaugua.................   4     1   Mm2
......................................................................uaaaugcauuaucugguCugua.................   20    1   Mm2
......................................................................uaaaugcauuaucugguaugCa.................   3     1   Mm2
......................................................................uCaaugcauuaucugguaugua.................   1     1   Mm2
......................................................................uaaaugcauuaucugguGugua.................   15    1   Mm2
......................................................................uaaaugcaCuaucugguaugua.................   1     1   Mm2
......................................................................uaaaugcGuuaucugguaugua.................   1     1   Mm2
......................................................................uaaauNcauuaucugguaugua.................   1     1   Mm2
......................................................................uaaaugcauuUucugguaugua.................   3     1   Mm2
......................................................................uaaaugcauuaucCgguaugua.................   1     1   Mm2
......................................................................uaaaCgcauuaucugguaugua.................   1     1   Mm2
......................................................................uaaaugcauAaucugguaugua.................   1     1   Mm2
......................................................................uaaaugcauuaucugguUuguaa................   1     1   Mm2
......................................................................uaaauNcauuaucugguauguaa................   1     1   Mm2
......................................................................uaaaugcauuaucuggAauguaa................   1     1   Mm2
......................................................................uaaaugcauuaucugguauguaa................   342   0   Mm2
......................................................................uaaaugcauuCucugguauguaa................   1     1   Mm2
......................................................................Aaaaugcauuaucugguauguaa................   1     1   Mm2
......................................................................uaaaugcauuaucugguauguaU................   34    1   Mm2
......................................................................uaaaugcauuGucugguauguaa................   2     1   Mm2
......................................................................uaaaugcauuaucuggGauguaa................   1     1   Mm2
......................................................................uaaaugcauuaucugguCuguaa................   1     1   Mm2
......................................................................uaaaugcauuaucugguauguaaU...............   2     1   Mm2
......................................................................uaaaugcauuaucugguauguaaA...............   3     1   Mm2
......................................................................uaaaugcauuaucugguauguaaUu..............   1     1   Mm2
.......................................................................aaaugcauuaucugguaugu..................   3     0   Mm2
.......................................................................Uaaugcauuaucugguaugua.................   1     1   Mm2
.......................................................................aaaugcauuaucugguaugua.................   4     0   Mm2
.......................................................................aaaugcauuaucugguauguaac...............   1     0   Mm2
........................................................................aaugcauuaucugguaGgua.................   1     1   Mm2
.........................................................................augcauuaucugguaugua.................   1     0   Mm2

..................................cguaucagaggugcauuugc.......................................................   26    0   Mm1
..................................cguaucagaggugcauuugca......................................................   16    0   Mm1
..................................cguaucagaggugcauuAgca......................................................   1     1   Mm1
.....................................................................Cuaaaugcauuaucugguaugua.................   1     1   Mm1
......................................................................uaaaugcauuaucuggua.....................   4     0   Mm1
......................................................................uaaaugcauuaucugguau....................   64    0   Mm1
......................................................................uCaaugcauuaucugguaug...................   1     1   Mm1
......................................................................uaaaugcauuaucugguauA...................   1     1   Mm1
......................................................................uaaaugcauuCucugguaug...................   3     1   Mm1
......................................................................uaaaugcauuUucugguaug...................   1     1   Mm1
......................................................................uUaaugcauuaucugguaug...................   2     1   Mm1
......................................................................uaaaugcauuaucugguUug...................   4     1   Mm1
......................................................................uaaaugcauuaucugguCug...................   6     1   Mm1
......................................................................uaaaugcCuuaucugguaug...................   1     1   Mm1
......................................................................uaaaugcauuaCcugguaug...................   1     1   Mm1
```

```
                                  Star                                  Mature
cuagauguuguuuuguugauaccuguggaggguucguaucagaggugcauuugcagugaaguauucuguguaaaugcauuaucugguauguaacucucgcauguugugc
......................................................................uaaaugcauuaucugguGug...................   4     1   Mm1
......................................................................uaaaugcauuGucugguaug...................   1     1   Mm1
......................................................................uaaaugcauuaucuggCaug...................   5     1   Mm1
......................................................................uaaaugcauuaucugguaug...................   854   0   Mm1
......................................................................uaaaugcauuaucugguauU...................   2     1   Mm1
......................................................................uaaaugcauuaucuggGaug...................   2     1   Mm1
......................................................................uaaaugcauuaucuggAaug...................   1     1   Mm1
......................................................................uaaaugcauuaucugAuaugu..................   1     1   Mm1
......................................................................uaaaugcGuuaucugguaugu..................   4     1   Mm1
......................................................................uaGaugcauuaucugguaugu..................   4     1   Mm1
......................................................................uaaaugcauuaucuggAaugu..................   15    1   Mm1
......................................................................uaaaugcaGuaucugguaugu..................   1     1   Mm1
......................................................................uaUaugcauuaucugguaugu..................   1     1   Mm1
......................................................................uaaaugcauuaucugguaCgu..................   1     1   Mm1
......................................................................uaaaugcUuuaucugguaugu..................   1     1   Mm1
......................................................................Aaaaugcauuaucugguaugu..................   1     1   Mm1
......................................................................uaaaugcauuaucugguauCu..................   1     1   Mm1
......................................................................uaaauAcauuaucugguaugu..................   1     1   Mm1
......................................................................uaaaugcauuaucuggGaugu..................   18    1   Mm1
......................................................................uaaaugcaAuaucugguaugu..................   1     1   Mm1
......................................................................uaaaugcauuCucugguaugu..................   7     1   Mm1
......................................................................uUaaugcauuaucugguaugu..................   4     1   Mm1
......................................................................uaaaCgcauuaucugguaugu..................   2     1   Mm1
......................................................................Gaaaugcauuaucugguaugu..................   1     1   Mm1
......................................................................uaaaugcauuaCcugguaugu..................   1     1   Mm1
......................................................................uaaaugcauuaucugguaGgu..................   1     1   Mm1
......................................................................uaaaugNauuaucugguaugu..................   2     1   Mm1
......................................................................uaaaugcauuaucugguUugu..................   24    1   Mm1
......................................................................uaaaugcauuaucGgguaugu..................   2     1   Mm1
......................................................................uaaaugcauuaucugguGugu..................   14    1   Mm1
......................................................................uaaaugcauuUucugguaugu..................   9     1   Mm1
......................................................................uaaaugUauuaucugguaugu..................   3     1   Mm1
......................................................................uaaaAgcauuaucugguaugu..................   1     1   Mm1
......................................................................uaaaugcauuaucAgguaugu..................   3     1   Mm1
......................................................................uaaaugcauuaucugguaugu..................   7427  0   Mm1
......................................................................uaaaugcauGaucugguaugu..................   1     1   Mm1
......................................................................uaaaugcauuaucugguaugG..................   12    1   Mm1
......................................................................Naaaugcauuaucugguaugu..................   4     1   Mm1
......................................................................uaaaugcauuGucugguaugu..................   11    1   Mm1
......................................................................uaCaugcauuaucugguaugu..................   1     1   Mm1
......................................................................uaaGugcauuaucugguaugu..................   1     1   Mm1
......................................................................uGaaugcauuaucugguaugu..................   5     1   Mm1
......................................................................uaaaugcauuaucuggCaugu..................   38    1   Mm1
......................................................................uCaaugcauuaucugguaugu..................   1     1   Mm1
......................................................................uaaaugcauuaucugguCugu..................   33    1   Mm1
......................................................................uaaauUcauuaucugguaugu..................   3     1   Mm1
......................................................................uaaaugcauuaucugguauguG.................   5     1   Mm1
......................................................................uaaGugcauuaucugguaugua.................   1     1   Mm1
......................................................................Aaaaugcauuaucugguaugua.................   1     1   Mm1
......................................................................uaaaugcauuaucugguaugua.................   6809  0   Mm1
......................................................................uaaaugcaGuaucugguaugua.................   1     1   Mm1
......................................................................uaaauUcauuaucugguaugua.................   1     1   Mm1
......................................................................uaaaugcauuaucugguUugua.................   13    1   Mm1
......................................................................uaaaugcauuaucAgguaugua.................   1     1   Mm1
......................................................................uaaaugcGuuaucugguaugua.................   3     1   Mm1
......................................................................Naaaugcauuaucugguaugua.................   8     1   Mm1
......................................................................uaaaugcauuaucGgguaugua.................   1     1   Mm1
......................................................................uaaaugcauuaucuggGaugua.................   18    1   Mm1
......................................................................uaaaugcUuuaucugguaugua.................   3     1   Mm1
......................................................................uUaaugcauuaucugguaugua.................   5     1   Mm1
......................................................................uaaauAcauuaucugguaugua.................   1     1   Mm1
......................................................................uaaaugcauuaucuggAaugua.................   14    1   Mm1
......................................................................uaaaugcauuaucugguGugua.................   25    1   Mm1
......................................................................uaaaugcauuaucugguauAua.................   1     1   Mm1
......................................................................uaUaugcauuaucugguaugua.................   1     1   Mm1
......................................................................uaaaugcauuaucugguauUua.................   1     1   Mm1
......................................................................uaaaugcauuaucugguaugCa.................   1     1   Mm1
......................................................................uaGaugcauuaucugguaugua.................   5     1   Mm1
......................................................................uaaaugcauuGucugguaugua.................   9     1   Mm1
......................................................................uaaaugcauuUucugguaugua.................   9     1   Mm1
```

Star **Mature**

```
cuagauguuguuuuguugauaccuguggaggguucguaucagaggugcauuugcagugaaguauucuguguaaaugcauuaucugguauguaacucucgcauguugugc
......................................................................uaaaugcauuaAcugguaugua.................   1     1   Mm1
......................................................................uaaaugcauuaucugguCugua.................   27    1   Mm1
......................................................................uGaaugcauuaucugguaugua.................   4     1   Mm1
......................................................................uaaaugcauuauUugguaugua.................   1     1   Mm1
......................................................................uaaaugcauuCucugguaugua.................   5     1   Mm1
......................................................................uaaaugcauuaucugguaugGa.................   1     1   Mm1
......................................................................uaaaugcauGaucugguaugua.................   1     1   Mm1
......................................................................uaaaugcauuaucuggCaugua.................   28    1   Mm1
......................................................................uaaaugcauCaucugguaugua.................   1     1   Mm1
......................................................................uaaaugUauuaucugguaugua.................   1     1   Mm1
......................................................................Caaaugcauuaucugguaugua.................   1     1   Mm1
......................................................................uaaauCcauuaucugguaugua.................   1     1   Mm1
......................................................................uaaaugcauuaucCgguaugua.................   3     1   Mm1
......................................................................uaaaugcauuaucugUuaugua.................   2     1   Mm1
......................................................................uaaaugcauuaucugguauguU.................   171   1   Mm1
......................................................................uaaaugcauuaucugguauguaU................   72    1   Mm1
......................................................................uaaaugcauuaucugguUuguaa................   5     1   Mm1
......................................................................uaaaugcauuCucugguauguaa................   2     1   Mm1
......................................................................uaaaugcauuaucuggGauguaa................   2     1   Mm1
......................................................................uaaaugcauuaucuggCauguaa................   3     1   Mm1
......................................................................uaaaugcauuaucugguGuguaa................   2     1   Mm1
......................................................................uaaaugcGuuaucugguauguaa................   1     1   Mm1
......................................................................uaaaAgcauuaucugguauguaa................   1     1   Mm1
......................................................................uaaaugcauuaucugguauguaa................   830   0   Mm1
......................................................................uaaaugcauuaucugguauguaG................   29    1   Mm1
......................................................................uaaaugcauuaucugguCuguaa................   6     1   Mm1
......................................................................uaaaugcauuaucugguauguaac...............   3     0   Mm1
......................................................................uaaaugcauuaucugguauguaaU...............   7     1   Mm1
......................................................................uaaaugcauuaucugguauguaaA...............   15    1   Mm1
.......................................................................aaaugcauuaucugguaug...................   1     0   Mm1
.......................................................................Uaaugcauuaucugguaugu..................   1     1   Mm1
.......................................................................Uaaugcauuaucugguaugua.................   1     1   Mm1
.......................................................................aaaugcauuaucugguaugua.................   6     0   Mm1
.......................................................................aaaugcauCaucugguaugua.................   1     1   Mm1
...........................................................................gcauuaucugguauguaG................   1     1   Mm1
...........................................................................gcauuaucugguauguaU................   6     1   Mm1
```
